# A Putative Mitochondrial Iron Transporter MrsA in *Aspergillus fumigatus* Plays Important Roles in Azole-, Oxidative Stress Responses and Virulence

**DOI:** 10.3389/fmicb.2016.00716

**Published:** 2016-05-12

**Authors:** Nanbiao Long, Xiaoling Xu, Hui Qian, Shizhu Zhang, Ling Lu

**Affiliations:** Jiangsu Key Laboratory for Microbes and Functional Genomics, Jiangsu Engineering and Technology Research Center for Microbiology, College of Life Sciences, Nanjing Normal UniversityNanjing, China

**Keywords:** *Aspergillus fumigatus*, mitochondrial iron transporter, iron homeostasis, oxidative stresses, ROS, histidine residues, azole stresses

## Abstract

Iron is an essential nutrient and enzyme co-factor required for a wide range of cellular processes, especially for the function of mitochondria. For the opportunistic fungal pathogen *Aspergillus fumigatus*, the ability to obtain iron is required for growth and virulence during the infection process. However, knowledge of how mitochondria are involved in iron regulation is still limited. Here, we show that a mitochondrial iron transporter, MrsA, a homolog of yeast Mrs4p, is critical for adaptation to iron-limited or iron-excess conditions in *A. fumigatus*. Deletion of *mrsA* leads to disruption of iron homeostasis with a decreased *sreA* expression, resulted in activated reductive iron assimilation (RIA) and siderophore-mediated iron acquisition (SIA). Furthermore, deletion of *mrsA* induces hypersusceptibility to azole and oxidative stresses. An assay for cellular ROS content in *ΔmrsA* combined with rescue from the *mrsA*-defective phenotype by the antioxidant reagent L-ascorbic acid indicates that the increased sensitivity of *ΔmrsA* to the azole itraconazole and to oxidative stress is mainly the result of abnormal ROS accumulation. Moreover, site-directed mutation experiments verified that three conserved histidine residues related to iron transport in MrsA are required for responses to oxidative and azole stresses. Importantly, *ΔmrsA* causes significant attenuation of virulence in an immunocompromised murine model of aspergillosis. Collectively, our results show that the putative mitochondrial iron transporter MrsA plays important roles in azole- and oxidative-stress responses and virulence by regulating the balance of cellular iron in *A. fumigatus*.

## Introduction

In all eukaryotic systems, iron is an essential element that is required for biological processes such as electron transport, heme and iron-sulfur cluster synthesis, DNA repair and other functions. For fungal pathogens, iron acquisition is a critical factor for virulence during the process of infection because the host or competitor always tightly sequesters the available iron and forms an essentially iron-free environment (Jung et al., 2010; Chen et al., 2011; Lopez-Berges et al., 2012; Moore, 2013). Thus, as an environmental pathogen, *A. fumigatus* has developed a complicated and effective iron homeostasis regulation system to survive in its ecological niches. Previous studies have shown that there are two high-affinity iron uptake systems, a reductive iron assimilation (RIA) system and a siderophore-mediated iron acquisition system (SIA), in *A. fumigatus* (Schrettl et al., 2004; Moore, 2013). The RIA system is composed of a putative ferroxidase, FetC, and an iron permease, FtrA, whereas the SIA consists of the iron chelator (siderophore) and its transporter MirB (Raymond-Bouchard et al., 2012; Moore, 2013). The first step in the synthesis of the siderophore is catalyzed by N5-ornithine monooxygenase, which is encoded by the *sidA* gene. The defect in siderophore synthesis caused by deletion of *sidA* results in elimination of the siderophores and absolute avirulence in a mouse model of pulmonary aspergillosis in *A. fumigatus* (Hissen et al., 2005). Thus, SIA is considered of great importance to the survival of *A. fumigatus* in the host.

In contrast, iron uptake in *A. fumigatus* is not “the more the better” because excess iron accumulation can produce noxious reactive oxygen species through the Haber–Weiss/Fenton reaction, resulting in damage to cellular proteins, DNA and cell membranes (Halliwell et al., 1984; Halliwell and Gutteridge, 1992). Therefore, to protect itself from damage due to abnormal iron accumulation, *A. fumigatus* has evolved two important, functionally opposed transcription factors, SreA and HapX, which regulate the iron homeostasis (Schrettl and Haas, 2011; Haas, 2012). Under conditions of iron sufficiency, highly expressed SreA is capable of repressing both the RIA and the SIA systems and thereby avoiding iron overload, whereas during iron starvation SreA is inactivated and HapX is highly expressed, repressing the iron-consuming pathway and promoting iron uptake (Schrettl et al., 2008, 2010). Nevertheless, it has recently been reported that HapX also functions under conditions of iron excess by causing the vacuolar iron transporter CccA to store iron within the vacuole (Gsaller et al., 2014). This information suggests that our knowledge of iron regulatory mechanisms in *A. fumigatus* is still limited.

Mitochondria play a key role in cellular iron homeostasis because they carry out the iron-consuming processes of heme and iron-sulfur cluster synthesis, which are involved in many important biological processes. Previous studies have verified that iron, as the substrate for these processes, must be imported into mitochondria by transporters (Paradkar et al., 2009; Chen and Paw, 2012). The budding yeast *Saccharomyces cerevisiae* contains two mitochondrial high affinity iron transporters ScMrs3p and ScMrs4p, which are located in the mitochondrial inner membrane and belong to the mitochondrial solute carrier family (Foury and Roganti, 2002; Muhlenhoff et al., 2003). Homologs of these genes are found in almost all eukaryotes, and mutations in these genes result in defective mitochondrial iron homeostasis in a wide range of species, including mammalian *Mus musculus*, the fungal pathogen *Candida albicans*, and others (Paradkar et al., 2009; Xu et al., 2012). Interference with the functions of the *Scmrs3* and *Scmrs4* homologs *mfrn1* and/or *mfrn2* in *M. musculus* by RNAi results in decreased mitochondrial iron accumulation and defective heme and iron-sulfur cluster synthesis (Paradkar et al., 2009). In comparison, in the fungal pathogen *C. albicans*, deletion of the putative mitochondrial iron transporter *Camrs4* inhibits cell growth, increases cellular iron content and produces a phenotype of hypersensitivity to oxidants (Xu et al., 2012). Moreover, a recent study has shown that three highly conserved histidine residues (His48 (H1), His105 (H2) and His222 (H5) in ScMrs3p or His38, His95 and His212 in ScMrs4p) are important for the function of ScMrs3p or ScMrs4p gene product in iron import, indicating that imidazole groups contained within the protein’s histidine residues act as the major iron ligands (Brazzolotto et al., 2014). Thus, deletion of *Scmrs3* and *Scmrs4* severely disrupts mitochondrial and cellular iron homeostasis by reducing cytosolic and mitochondrial iron acquisition. On the other hand, decreased cytosolic iron acquisition activates Aft1p, an iron-sensing transcription factor that is involved in iron utilization and homeostasis, which results in increased iron uptake by increasing the expression of the high-affinity iron transport systems (Li and Kaplan, 2004).

In contrast to the well-known mechanism of mitochondrial iron transport and regulation in yeasts, *A. fumigatus* lacks specific iron uptake systems from host iron sources such as heme, ferritin and transferrin, indicating that this filamentous fungal pathogen of humans, which is able to cause life-threatening invasive disease, especially in immunocompromised patients, may possess a unique mechanism for mitochondrial and cellular iron homeostasis (Schrettl et al., 2004). However, our knowledge of intracellular iron transport and regulation in *A. fumigatus*, especially with respect to the function of mitochondria, is still limited.

In this study, we found that deletion of the putative iron transporter *mrsA*, a homolog of *mrs3* and *mrs4* of *S. cerevisiae*, results in disruption of iron homeostasis in *A. fumigatus*. Assays of cellular ROS content in *ΔmrsA* combined with experiments demonstrating rescue of the *mrsA*-defective phenotype by the antioxidant reagent L-ascorbic acid indicate that the increased sensitivity of *ΔmrsA* to the azole itraconazole and to oxidative stress results primarily from abnormal ROS accumulation.

## Materials and Methods

### Strains, Media, and Culture Condition

All strains of *A. fumigatus* used in this study were given in **Table 1**. The A1160 (*Δku80*, *pyrG*) was used as the background strain. The media that used were as follows: YAG (2% glucose, 0.5% yeast extract, 1 ml/L 1000× trace elements); YUU (YAG supplemented with 5 mM uridine and 10 mM uracil) (Jiang et al., 2014). For anti-fungal assay, *A. fumigatus* strains were grown on the YAG medium containing different concentrations of H_2_O_2_, menadione and itraconazole, respectively. To test the iron sensitivity, the iron chelator bathophenanthroline disulfonic acid (disodium salt) (BPS) or FeCl_3_ was supplemented in the YAG medium. For the plate pointed assay, 2-μl slurry of spores from the different concentration of stock suspensions (10^7^, 10^6^,10^5^/ml) were spotted onto YAG or YUU. All strains were incubated at 37°C for 1.5–2 days (Jiang et al., 2014).

**Table 1:** *Aspergillus fumigatus* strains used in this study.

Strain	Genotype	Reference or source
A1160	*Δku80*, *pyrG*	FGSC
A1160^C^	*Δku80*, A1160::*pyrG*	Jiang et al., 2014
LN01	*Δku80*, *pyrG*, *ΔmrsA::pyr4*	This study
LN02	*Δku80*, *pyrG*, *ΔmrsA::pyr4*, *mrsA*, *hph*	This study
LN03	*Δku80*, *pyrG*, *mrsA::GFP::pyrG*	This study
LN04	*Δku80*, *pyrG*, *ΔmrsA::pyr4*, *mrsA^H38A^*, *hph*	This study
LN05	*Δku80*, *pyrG*, *ΔmrsA::pyr4*, *mrsA^G60A^*, *hph*	This study
LN06	*Δku80*, *pyrG*, *ΔmrsA::pyr4*, *mrsA^H96A^*, *hph*	This study
LN07	*Δku80*, *pyrG*, *ΔmrsA::pyr4*, *mrsA^H214A^*, *hph*	This study
LN08	*Δku80*, *pyrG*, *ΔmrsA::pyr4*, *gpdA(p)::Scmrs3*, *hph*	This study
LN09	*Δku80*, *pyrG*, *ΔmrsA::pyr4, gpdA(p)::Scmrs4*, *hph*	This study

### Knockout Cassette, Plasmid Construction and Transformation

The total primers used in this study are shown in Supplementary Table S1. For construction of *mrsA* deletion, the fusion PCR was used as previously described (Szewczyk et al., 2006). Briefly, approximately 1 kb of the upstream and downstream flanking sequences of the *mrsA* gene were amplified using the primers mrsA P1/P3 and mrsA P4/P6, respectively. The selection marker *pyr4* approximately 2 kb in size originally from *Neurospora crassa* was amplified from the plasmid pAL5 using the primer pair pyr4 F/R. Lastly, the three aforementioned PCR products were then fused with the primers mrsA P2/P5, and then transformed to relative receipt strain.

For complementation of *mrsA* null mutant, a full-length *mrsA* gene was amplified using the primer pair mrsA F/R, which includes the native promoter, the 5′UTR, the *mrsA* gene coding sequence and the 3′UTR. This DNA fragment was subcloned into pEASY-Blunt zero (TransGen Biotech) according to the manufacturer’s directions. Next, a 4-kb fragment containing the hygromycin B resistance gene *hph* was amplified with the primers hph-*Spe*I F/R from the plasmid pAN7-1 and inserted into the *Spe*I site of the pEASY-Blunt zero vector as a selectable marker.

To constitutively express *Scmrs3* and *Scmrs4* in the background of Δ*mrsA*, the hygromycin B resistance gene *hph* was amplified with primers hph-*Not*I F and hph-*Spe*I R and then cloned into the *Not*I and *Spe*I site of pBARGPE to generate pBARGPE-1 (Song et al., 2015). The open reading frame (ORF) of *Scmrs3p* and *Scmrs4p* was amplified from the genomic DNA of *S. cerevisiae* S288c with primers Scmrs3/4 *Cla*I F/R and subcloned into the *Cla*I site of pBARGPE-1 to generate a constitutively expressed plasmid.

To create an mrsA-GFP cassette, approximately the 1.7 kb upstream sequence (except the termination codon) and the 1.6 kb downstream sequence (including the termination codon) of *mrsA* were amplified using mrsA-gfp P1/P3 and mrsA-gfp P4/P6, respectively. The fragments that contain GFP and the selection marker *AfpyrG* were amplified from the plasmid pFNO3 using the primers gfp-pyrG F/R. Those three fragments were then fused with primers mrsA-gfp P2/P5 to construct the GFP-tagging cassette.

For site-directed mutagenesis, the following strategy was employed. Briefly, complementary primers approximately 30 bp in length that includes the desired mutation in the center position were designed and synthesized. The plasmid harboring *mrsA* wild-type gene was used as a template. Each desired mutation was amplified with respective primers and the resulting PCR products were treated with *Dpn*I to digest the template plasmid and then transformed into *Escherichia coli*. All the plasmids that contained the desired mutation were sequenced to ensure the predicted site-directed mutagenesis happened.

Transformation of *A. fumigatus* was performed as described previously (Szewczyk et al., 2006). For the marker used with *hph*, 200 μg/ml hygromycin B was added to the medium for the selection of transformants.

### Western Blotting Analysis

For Western blots, mycelia were ground in liquid nitrogen and suspended in pre-cold protein extraction buffer (50 mM HEPES pH 7.4, 137 mM KCl, 10% glycerol 1 mM EDTA, 1 μg/ml pepstatin A, 1 μg/ml leupeptin and 1 mM PMSF) (Cai et al., 2015). Samples were incubated on ice and vortex 30 s every 5 min for three times. Cell debris were removed by centrifugation with 13000 × *g*, 4°C, 10 min. The concentration of protein was measured by Bio-Rad protein assay kit. GFP-tag was detected with the anti-GFP mouse monoclonal antibody (Roche) at 1:3,000. Anti-actin antibody used in this study was purchased from ICN Biomedicals Inc. with 1:20,000. Peroxidase-conjugated goat anti-mouse and rabbit IgG was used at a 1:4,000 dilution. Peroxidase activity was detected using ECL kit (Roche).

### Fluorescence Microscopy

To visualize the localization of MrsA-GFP, the relative strain was grown on coverslips in 3 ml YAG media for 18 h. The mitochondria dye- Mito-Tracker (Invitrogen) that dissolved in phosphate buffer saline (PBS) was used at a final concentration of 50 nM and incubated for 5 min at the room temperature. Images were captured using a Zeiss Axio imager A1 microscope (Zeiss, Jena, Germany) and the picture was managed with Adobe Photoshop.

### RNA Extraction for RT-PCR

For RT-PCR, total RNA was isolated from the fresh mycelium using TRIzol (Roche) as described by manufacturer’s instructions. The digestion of genomic DNA and the synthesis of cDNA were performed using HiScript^®^ II Q RT SuperMix for qPCR kit (Vazyme) as its instruction book. qRT-PCR was executed by ABI One-step fast thermocycler (Applied Biosystems) with SYBR Premix Ex Taq^TM^ (TaKaRa).

### Reactive Oxygen Species Measurement

The production of reactive oxygen species was measured as described by Li et al. (2010). Briefly, 10^7^ spores of strains in 100 ml YAG media were incubated at 37°C for 18 h with shaking at 220 rpm. 2′,7′-Dichlorodihydrofluorescein diacetate (H2DCFDA) (Invitrogen) with a final concentration of 15 μM was added to the medium and then incubated at 37°C for 1 h. After that, the mycelia were harvested and washed for three times with the distilled water to remove extracellular H2DCFDA. The filtered mycelia were then ground in liquid nitrogen and incubated in PBS. The resulting supernatant was collected by centrifugation at 15,000 × *g* and 4°C for 10 min. Fluorescence was measured using SpectraMax M2 (Molecular Devices, USA) with an excitation wavelength of 504 nm and an emission wavelength of 524 nm. The fluorescence intensity was normalized to the protein concentration of the sample, which was measured using a Bio-Rad protein assay kit.

### Extraction and Analysis of Whole Cell Ergosterol

For total ergosterol extraction from *A. fumigatus* strains, 10^8^ spores of strains in 100 ml YAG media were incubated at the speed of 220 rpm at 37°C for 24 h. Mycelia were obtained via filtration with gauze and washed three times with distilled water and lyophilized. About 200 mg dry mycelia were ground and extracted as described previously (Alcazar-Fuoli et al., 2008; Yasmin et al., 2012).

### Flow Cytometry

To measure the cellular accumulation of azole-mimicked reagent Rh123, the method was followed as described previously (Clark et al., 1996; Mukherjee et al., 2003). Briefly, 10^7^ spores of parental wild type and mutant strains were incubated at 37°C for 4 h in a rotary shaker at the speed of 220 rpm. A final concentration of 10 μM Rh123 was added to the conidial suspension and incubated at 37°C for another 1 h. Spores were obtained by centrifuge at 5000 × *g* for 5 min, and then washed three times with PBS to remove extra-cellular Rh123. The fluorescent signal was quantified using a Becton Dickinson FACSort (fluorescence activated cell sorter) with 488 nm as the excitation wavelength and 546 nm as the emission wavelength, which was adjusted to a fixed channel using standard Brite Beads (Coulter, Miami, FL, USA) prior to fluorescence detection.

### Cellular Drug Detection

To detect cellular drug accumulation, the method was used as described previously (Zheng et al., 2015; Kretschmann et al., 2016) but modified to some extent. Briefly, approximately 10^7^ spores of strains in 100 ml YAG media were incubated at 37°C for 18 h at 220 rpm. A final concentration of 1 mg/ml of the anti-fungal drug ITZ was added to the medium, and the cultures were incubated at 37°C for an additional 1 h. Mycelia were harvested and washed for three times with distilled water to remove extracellular ITZ. Fifty milligram of lyophilized mycelia was homogenized with 1 ml methanol in the presence of ceramic beads. Then, the cell debris and ceramic beads were removed by centrifugation at 13,000 × *g* for 10 min. The supernatant containing the antifungal drug ITZ was analyzed using HPLC on an AQ-C18 column (250 mm by 4.6 mm, 5 μm) with a flow rate of 1 ml/min of methanol at 265 nm.

### Virulence Assay

Virulence assays in this study were performed as described previously (Jiang et al., 2014). Briefly, 6–8-week-old ICR male mice were immunosuppressed on day -3 and -1 with cyclophosphamide (150 mg/kg) and on day -1 with hydrocortisone acetate (40 mg/kg). On day 0, mice were anesthetized with pentobarbital sodium, and infected intratracheally with 50 μl slurry that contain 10^6^ conidia or 50 μl PBS as the control, according to the methods developed by Li et al. (2014) and Zhang et al. (2015). Cyclophosphamide (75 mg/kg) was injected every 3 days to maintain immuosupression. The mortality was monitored during 14 days in total after inoculation. For histopathological analysis, lungs were removed from the dead mice and maintained in 4% formaldehyde (v/v) before periodic acid-schiff staining. All animal experiments in this study were performed according to the Guide for the Care and Use of Laboratory Animals of the U.S. National Institutes of Health. The animal experimental protocol was approved by the Animal Care and Use Committee of Nanjing Normal University, China (permit no. 2090658) according to the governmental guidelines for animal care.

## Results

### Identification of MrsA in *A. fumigatus*

To identify putative homologs of Mrs3p and Mrs4p, which act as mitochondrial iron transporters in *S. cerevisiae*, in *A. fumigatus*, the amino acid sequences of Mrs3p and Mrs4p were used as a queries in BLASTP analysis of the genome database of *A. fumigatus* (Muhlenhoff et al., 2003). When using Mrs3p as a query, the BLASTP result showed the top two homolog candidates were AFUB_078550 (*e*-value 4e-87, identity 53%) and AFUB_090970 (*e*-value 3e-23, identity 28%), while using Mrs4p as a query, the result showed AFUB_078550 (*e*-value 5e-80, identity 49%) and AFUB_031430 (*e*-value 2e-24, identity 26%) were the top hits, which indicates that AFUB_078550 is the best match to both Mrs3p and Mrs4p of *S. cerevisiae*. Subsequent BLASTP analysis of the genome database of *S. cerevisiae* using AFUB_078550 as a query identified ScMrs3p and ScMrs4p as possible homologs. This suggests that AFUB_078550, here referred to as MrsA, might be the potential homolog of both ScMrs3p and ScMrs4p in *A. fumigatus*. To address whether MrsA is the only homolog in *A. fumigatus*, we blasted MrsA in the database of *A. fumigatus*, the result showed that except for MrsA itself (AFUB_078550, *e*-value 0, identity 100%), other two top hits are AFUB_090800 (*e*-value 1e-29, identity 31%) and AFUB_093110 (*e*-value 9e-25, identity 30%). Apparently, MrsA is the only homolog of ScMrs3p and ScMrs4p in *A. fumigatus*.

Based on its predicted sequence, MrsA contains 305 amino acid residues and shares 53% and 49% identity with *S. cerevisiae* Mrs3p and Mrs4p, respectively. To further investigate MrsA, an additional BLASTP analysis was performed using the amino acid sequence of MrsA as a query. As shown in Supplementary Figure S1A, MrsA homologs are conserved in selected organisms such as *S. cerevisiae*, *Homo sapiens*, *Arabidopsis thaliana*, *Caenorhabditis elegans*, *Schizosaccharomyces pombe*, *Oryza sativa*, *Drosophila melanogaster* and *Danio rerio*. Given the similarity Blast analysis of *C. albicans* (Xu et al., 2012), *A. fumigatus* and *C. albicans* most likely possess a single Mrs3/4p homolog (Supplementary Figure S1B). To further analyze the MrsA homologs in other pathogenic fungi, we blasted 10 different pathogenic fungi, which randomly cover human and plant pathogens. With expected, all the selected pathogenic fungi only have one homolog of MrsA (Supplementary Figure S1C).

All the identified MrsA homologs contain six conserved transmembrane helices (H1–H6) and exhibit a tripartite structure. Each of the three tandem structures contains two helical regions approximately 100 amino acids in length and a conserved PX (D/E) X2 (K/R) motif (Brazzolotto et al., 2014). Three highly conserved histidine residues similar to those that serve as ligands in numerous iron-containing proteins and are considered to play important roles during iron import from the cytoplasm to mitochondria lie within H1, H2, and H5 of Mrs3p and Mrs4p. In *S. cerevisiae*, mutation of every one of the three conserved histidine residues affects the function of Mrs3p and Mrs4p and results in decreased iron import (Brazzolotto et al., 2014).

### MrsA Is a Putative Mitochondrial Iron Transporter in *A. fumigatus*

**FIGURE 1:**
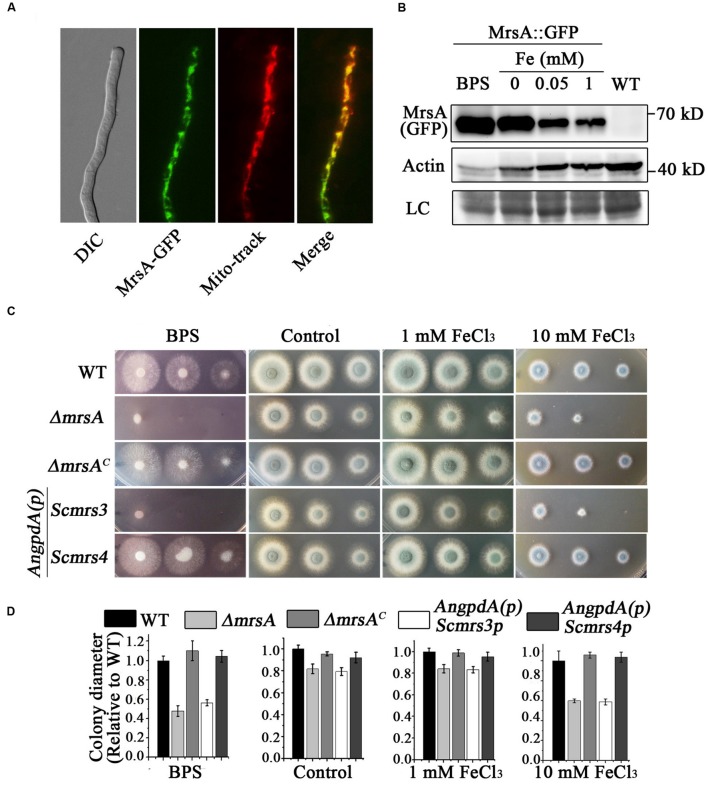
**MrsA is a putative mitochondrial iron transporter of yeast Scmrs4 homolog in *A. fumigatus*. (A)** GFP-tagged MrsA was located in mitochondria. Mito-tracker was used to visualize mitochondria. Scale bar = 10 μm. **(B)** Western blot analysis of MrsA-GFP in the condition of BPS or iron. The molecular mass of MrsA-GFP is 62 kD (27 kD GFP + 35 kD MrsA). Actin (42 kD) was used as the loading control. LC represent amido black staining. **(C)** Colony morphologies of parental wild type, *ΔmrsA, mrsA*-reconstituted (*ΔmrsA^C^*), and *Scmrs3*- and *Scmrs4*- reconstituted strains in the presence of 300 μM BPS and 1 or 10 mM Fe (FeCl_3_) in YAG media. *Scmrs3* and *Scmrs4*-reconstituted genes under the control of the *AngpdA* promoter were transformed into the *ΔmrsA* strain. **(D)** Quantifications of colony diameter for the indicated strains under the different treatment cultural condition.

To verify the subcellular localization of MrsA, we labeled the C terminus of MrsA with GFP under the control of its native promoter. The phenotype of MrsA::GFP under all tested conditions was similar to that of its parental wild-type strain, indicating that MrsA::GFP is fully functional. Western blot results showed a specific band of molecular size approximately 62 kD (including GFP, 27 kD), indicating that GFP-tagged MrsA was expressed at its predicted size (**Figure 1B**). As shown in **Figure 1A**, the MrsA-GFP fusion protein co-localized with the mitochondrial marker MitoTracker Red (Invitrogen), suggesting that MrsA is located in mitochondria. To address whether the expression of MrsA is affected by iron concentration, we tested the protein level of MrsA under the condition of iron starvation or iron-replete, respectively. As shown in **Figure 1B**, under the iron starvation condition, the expression of MrsA was obviously increased compared to that under the iron-replete condition, suggesting that MrsA function is particularly important during iron starvation.

To better understand whether MrsA is involved in iron transport in mitochondria, we generated an *mrsA* null mutant by homologous gene replacement. Diagnostic PCR analysis showed that *pyr4*, which was used as a selection marker, completely replaced the ORF of *mrsA*, suggesting that the ORF of *mrsA* was fully deleted (Supplementary Figure S2). As shown in **Figures 1C**,**D**, the colony growth of *mrsA* null mutant displayed slight differences compared to that of the wild-type parental strain (81.7% ± 4.4 in *mrsA* deletion versus 100% in parental strain) when conidia were inoculated in the rich medium YAG. However, when cultured in medium supplemented with the iron–specific chelator bathophenanthroline disulfonic acid (disodium salt) (BPS) at a concentration of 300 μM, *ΔmrsA* showed very severe colony growth defects with a tiny and fluffy colony phenotype, indicating that MrsA is required for hyphal growth and conidiation under conditions of iron depletion. In comparison, when iron (FeCl_3_) was added to the medium at a concentration of 10 mM, *ΔmrsA* showed a more sensitive phenotype than its parental wild type. Moreover, when a full-length *mrsA* gene was transformed into *ΔmrsA*, all tested colonies were restored to the phenotype of the parental wild type under the culture conditions described above, suggesting that the above-described colony defects are specifically due to loss of *mrsA*. Collectively, these data suggest that MrsA plays important roles in hyphal growth and conidiation both under conditions of iron depletion and under conditions of iron excess (**Figure 1C**).

Because the aforementioned data indicate that MrsA shares 53 and 49% identity, respectively, with Mrs3p and Mrs4p, proteins that act as mitochondrial iron transporters in *S. cerevisiae*, the three proteins may have conserved functions. This led us to explore whether introducing the ScMrs3p or ScMrs4p gene would eliminate the defects in the *ΔmrsA* mutant. To this purpose, we expressed Mrs3p and Mrs4p under the control of a constitutive *AngpdA* promoter in *ΔmrsA*. As shown in **Figure 1C**, Mrs4p, but not Mrs3p, was able to fully restore the colony defects of *ΔmrsA* under both iron depletion and iron excess conditions. This result strongly suggests that MrsA is a functional conserved homolog of Mrs4p.

### Deletion of *mrsA* Results in Significant Changes in the mRNA Levels of Genes Involved in Iron Regulation

**FIGURE 2:**
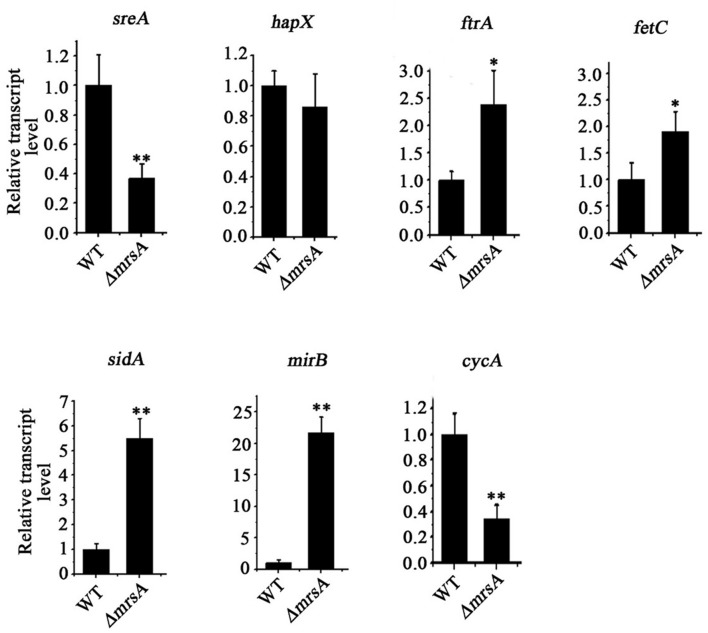
**Deletion of *mrsA* results in abnormal expression of iron homostasis regulation-related genes.** The indicated strains of *A. fumigatus* were incubated in YAG for 24 h at 37°C. The relative expressions of the indicated genes were determined by real-time PCR. ^∗^*P* < 0.05 and ^∗∗^*P* < 0.01 compared with parental wild type.

Based on our finding that loss of *mrsA* affects adaptation to both iron depletion and iron excess conditions, we designed experiments to test whether the major transcription factors *sreA* and *hapX* or genes related to iron regulation whose expression is controlled by these transcription factors are affected by loss of *mrsA*. As predicted, compared to the parental wild type, the mRNA expression level of *sreA* decreased significantly in *ΔmrsA*. In comparison, there was no detectable change in the mRNA level of *hapX* (**Figure 2**). We further analyzed the expression of *sreA*-regulated genes associated with RIA, including *ftrA* and *fetC*, and that of genes associated with SIA, including *sidA* and *mirB* (Schrettl et al., 2008). As shown in **Figure 2**, the mRNA levels of *ftrA*, *fetC*, *sidA* and *mirB*, especially *sidA* and *mirB*, were markedly increased in *ΔmrsA*, suggesting that deletion of *mrsA* activates the RIA and siderophore-mediated iron uptake systems. In contrast, the expression of iron-consuming pathway-related genes located in mitochondria, such as *cycA* (cytochrome C), which functions in respiration, was decreased significantly in *ΔmrsA* compared to the parental wild type strain (Schrettl et al., 2010). Those data indicate that the RIA and SIA were activated when *mrsA* was lost, whereas iron-consuming pathway-related genes located in mitochondria were repressed.

### MrsA Deficiency Leads to Increased Susceptibility to Oxidative Stress

**FIGURE 3:**
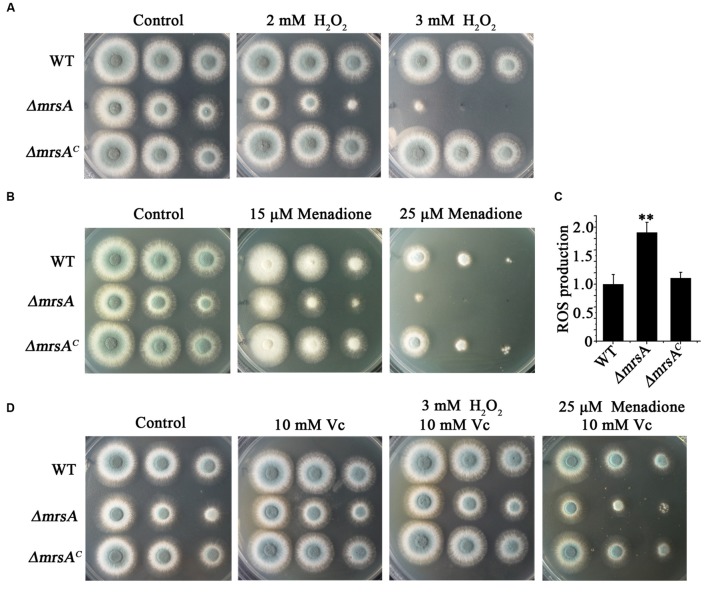
**MrsA deficiency leads to increased susceptibility to oxidative stress. (A,B)** Two microliters of DDW (double distill water) containing 10^4^, 10^3^ or 10^2^ conidia of each strain were used to inoculate YAG medium containing H_2_O_2_ (2 or 3 mM) or menadione (15 or 25 μM). **(C)** Reactive oxygen species (ROS) production of the parental wild-type strain, *ΔmrsA* and *ΔmrsA^C^*. The ROS contents of *ΔmrsA* and *ΔmrsA^C^* were normalized to that of the parental wild type (wild type = 1). ^∗∗^*P* < 0.01 compared with parental wild type and *ΔmrsA^C^*. **(D)** Serially diluted conidia of each strain were spotted onto YAG plates containing the ROS scavenger L-ascorbic acid sodium (Vc, 10 mM) and/or H_2_O_2_ (3 mM) and menadione (25 μM).

Previous studies have demonstrated that dysfunctions of mitochondria, especially dysfunctions induced by disturbed iron levels, lead to hypersensitivity to oxidative stress (Lin et al., 2011; Thomas et al., 2013; Kong et al., 2014). Therefore, we wondered whether mitochondrially localized MrsA affects the response of fungal cells to oxidative stress. To test this hypothesis, we compared the phenotypes of *ΔmrsA* and its parental wild type in the presence of H_2_O_2_. As shown in **Figure 3A**, *ΔmrsA* showed enhanced sensitivity to the addition of H_2_O_2_ to the medium_._ This sensitivity was dose-dependent such that the *mrsA* deletion mutant showed almost no detectable colony growth in the presence of 3 mM H_2_O_2_. When another oxidative reagent, menadione (25 μM), was added to the medium, a hypersensitivity phenotype of the *ΔmrsA* mutant was also observed compared to its parental wild type (**Figure 3B**). However, the complemented strain of *ΔmrsA* exhibited a phenotype similar to that of the parental strain under both H_2_O_2_- and menadione-added conditions, suggesting that the strain’s hypersensitivity to H_2_O_2_ resulted from the deletion of *mrsA*.

Next, we were curious as to whether the observed hypersensitivity of *ΔmrsA* to oxidative stress was due to an increased level of reactive oxygen species (ROS). To measure the production of ROS, the specific probe 2′,7′-dichlorodihydrofluorescein diacetate (H2DCFDA), which can be metabolized to a fluorescent form of 2′,7′-dichlorodihydrofluorescein (H2DCF) by ROS, was used. As shown in **Figure 3C**, ROS production in *ΔmrsA* was significantly higher than in the parental wild-type strain and in the complemented strain. To further test the possible relationship between H_2_O_2_ and menadione hypersensitivity and the increased ROS level in *ΔmrsA*, L-ascorbic acid sodium (Vc), an efficient ROS scavenger, was added to the medium. As shown in **Figure 3D**, compared to treatment with H_2_O_2_ (3 mM) only, L-ascorbic acid sodium (10 mM) almost completely restored the colony phenotype of *ΔmrsA* to that of the parental wild-type strain in the presence of H_2_O_2_. Similarity, compared to treatment with menadione (25 μM) only, addition of L-ascorbic acid sodium significantly rescued the colony defect phenotype of *ΔmrsA* induced by menadione. This suggests that the abnormal ROS level in the *mrsA* mutant is strongly related to its phenotype of hypersensitivity to oxidative stress.

### *mrsA* Deficiency Leads to Increased Sensitivity to Itraconazole

**FIGURE 4:**
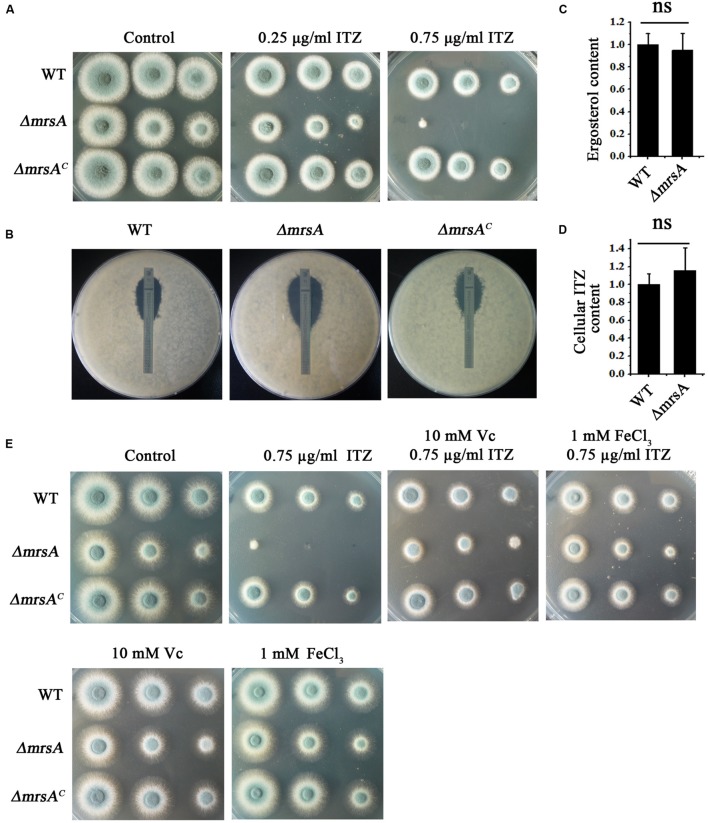
***ΔmrsA* shows increased susceptibility to the antifungal drug itraconazole (ITZ). (A)** Two microliters of DDW containing 10^4^, 10^3^, or 10^2^ conidia of each strain were used to inoculate YAG medium containing 0.25 or 0.75 μg ml^-1^ ITZ. Colony growth was compared to that obtained on YAG containing no drugs. **(B)** For each strain, 1 × 10^5^ conidia were mixed in YAG, and *E*-test strips of ITZ were placed on the plates. The MIC of *ΔmrsA* (0.38 μg ml^-1^) was significantly lower than that of the wild-type (1.0 μg ml^-1^) and *ΔmrsA^C^* (1.2 μg ml^-1^) strains. **(C,D)** Ergosterol production and intracellular ITZ accumulation of the parental wild-type strain and *ΔmrsA* were quantified using HPLC. Ergosterol and ITZ content of *ΔmrsA* was normalized to the level found in the parental strain (wild type = 1). **(E)** Serially diluted suspensions of conidia of each strain were spotted onto YAG plates containing the ROS scavenger L-ascorbic acid sodium (Vc, 10 mM) and/or ITZ (0.75 μg ml^-1^) and FeCl_3_ (1 mM).

Previous studies have indicated that the expression of mitochondria-related genes may affect the level of plasma membrane stress induced by antifungal azoles (Brun et al., 2003; Shingu-Vazquez and Traven, 2011; Thomas et al., 2013). Thus, we hypothesized that the susceptibility of the *mrsA* null mutant to the antifungal ITZ could be affected. When ITZ was added to the medium at a concentration of 0.75 μg/ml, *ΔmrsA* showed much more sensitivity than the parental wild-type strain, producing very small and sick colonies compared to those of the parental strain (**Figure 4A**). Commercial E-test strips, which indicate minimum inhibitory concentrations (MICs), showed that the value of MIC for *ΔmrsA* was 0.38 μg/ml, significantly lower than that of the parental wild-type (1.0 μg/ml) strain and of the complementary strain (1.2 μg/ml), suggesting that *ΔmrsA* is more sensitive to azole antifungals than both the parental wild type strain and the complementary strain *ΔmrsA^C^* (**Figure 4B**).

To identify the mechanism of azole susceptibility of *ΔmrsA*, we first examined whether the strains displayed any differences in ergosterol biosynthesis. The target protein of azole antifungal ITZ, 14-α sterol demethylase, is a key P450 enzyme that catalyzes the C-14 demethylation of lanosterol (Alcazar-Fuoli et al., 2008; Alcazar-Fuoli and Mellado, 2012). We compared the total ergosterol levels in the parental wild-type strain and the *mrsA* mutant by high-performance liquid chromatography (HPLC) analysis. The results showed no significant difference between the tested strains (**Figure 4C**). Next, we wondered whether the strains might differ in their intracellular accumulation of antifungal drugs, one of the main mechanisms of drug susceptibility across fungal pathogens (Wei et al., 2015). Using the fluorescent dye rhodamine 123 (Rh123), a drug molecule-mimicking substrate that is extruded from cells by transporters in an energy-dependent manner (Liu et al., 2015), we found significantly greater retention of Rh123 (*P* < 0.001) in the *ΔmrsA* mutant (73.61 ± 5.04 arbitrary units) than in the parental wild type strain (29.29 ± 6.28 arbitrary units) (Supplementary Figures S3A,B). This finding implies that *mrsA* deletion results in abnormal accumulation of Rh123. However, Rh123 has another function: monitoring changes in mitochondrial transmembrane potential that reflect the overall activity of mitochondria in the living cells (Ludovico et al., 2001). To assess whether the retention of Rh123 in *ΔmrsA* mimics the accumulation of antifungal drugs, we directly measured the intracellular accumulation of antifungal drugs by HPLC. HPLC analysis showed that the intracellular accumulation of ITZ by *ΔmrsA* and the parental wild type strain does not differ, suggesting that *ΔmrsA* hypersensitivity to ITZ did not result from differences in intracellular ITZ transport by the two strains (**Figure 4D**). Instead, the accumulation of Rh123 in *ΔmrsA* may be due to reduced energy production caused by dysfunction of mitochondria in the *mrsA* mutant. In *C. albicans*, it has been shown that endogenous ROS is an important mediator of the activity of the antifungal agent miconazole (Kobayashi et al., 2002). Our aforementioned data also show that loss of *mrsA* significantly increases cellular ROS production. Therefore, our results suggest that the hypersensitivity of *ΔmrsA* to ITZ may be related to cellular ROS production. To verify this point, we tested the growth of *ΔmrsA*, *ΔmrsA^C^* and the parental wild type strain in the presence of ITZ and L-ascorbic acid. Compared to ITZ alone, treatment with both ITZ and L-ascorbic acid dramatically reversed the phenotype of *ΔmrsA* to that of the parental wild type and the complemented strain (**Figure 4E**). Moreover, it was reported that ascorbic acid could improve the iron uptake by reducing iron and fueling the low-affinity iron uptake (Eisendle et al., 2003). We next tested whether the addition of iron would contribute to the resistance of ITZ in *A. fumigatus*. Interestingly, as shown in **Figure 4E**, compared to treatment with ITZ (0.75 μg/ml) only, extra addition of iron (1 mM) significantly rescued the colony growth defect phenotype of *ΔmrsA* induced by ITZ, indicating iron has a similar function to that of L-ascorbic acid. Collectively, our data suggest that increased cellular ROS production or the dysfunction of iron assimilation is likely the reason for the result of hypersensitivity in *ΔmrsA* induced by the anti-fungal compound ITZ.

### Three Conserved Histidine Residues Related to Iron Transport in MrsA Are Required for Response to Oxidative and Azole Stresses

**FIGURE 5:**
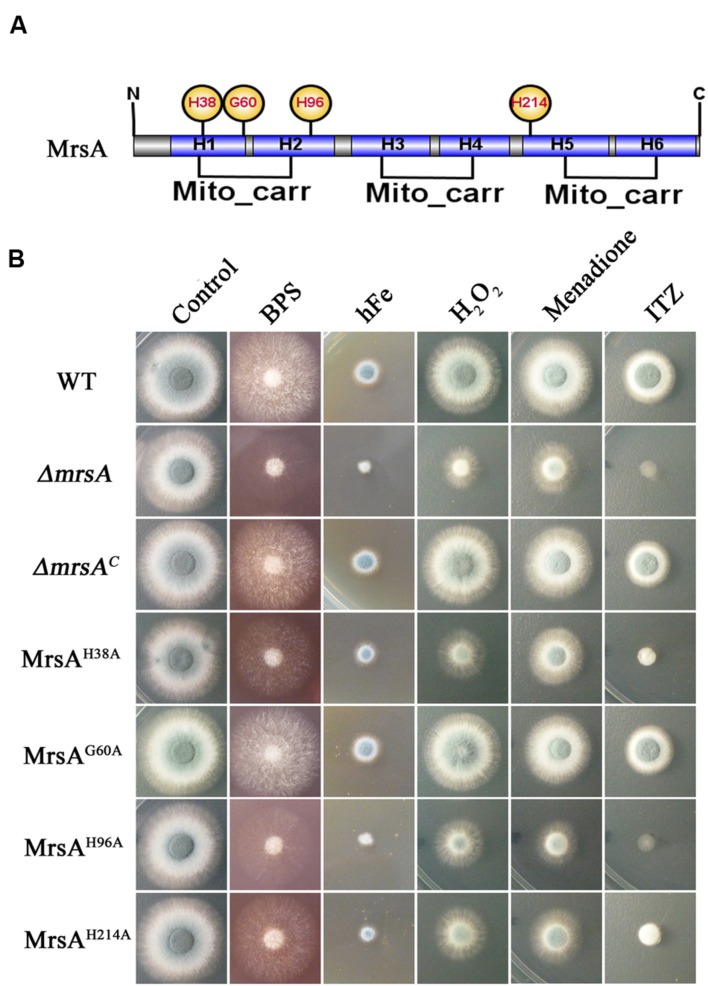
**Three conserved histidine residues in MrsA are required for the response to oxidative and azole stresses. (A)** Schematic view of the MrsA point mutation. The His residues (H) of MrsA located at positions 38 (H1), 96 (H2), and 214 (H5) and a glycine residue (G) located at position 60 (H1) were mutated to alanine (A). The glycine residue was mutated as a control. **(B)** Conidia of each strain were serially diluted and spotted onto YAG plates in the presence of 300 μM BPS, 10 mM Fe (FeCl_3_), 3 mM H_2_O_2_, 25 μM menadione and 0.75 μg ml^-1^ ITZ.

To further dissect the molecular characteristics of MrsA related to iron homeostasis and to stress responses, we used site-directed mutagenesis to mutate the conserved histidine (His) residues of MrsA located at positions 38 (H1), 96 (H2), and 214 (H5), based on information from ScMrs4p indicating that the three His residues are related to iron transport in yeast (Brazzolotto et al., 2014). A glycine residue located at position 60 (H1) in MrsA that is not conserved was mutated as a control (**Figure 5A**). Compared to the parental wild-type and the complemented strains, transformants that introduced MrsA^H38A^, MrsA^H96A^ and MrsA^H214A^ to the *ΔmrsA* strain showed obvious colony growth defects under iron-limiting and iron-excess conditions [produced by BPS (300 μM) and FeCl_3_, respectively], whereas transformed MrsA^G60A^ showed a phenotype similar to that of the parental wild-type strain. This suggests that the histidine residues located at positions 38 (H1), 96 (H2), and 214 (H5) of the MrsA sequence are required for the function of MrsA both under conditions of iron starvation and under conditions of iron excess (**Figure 5B**).

To further verify whether site-directed mutation of the conserved histidine (His) residues of MrsA affects the stress response induced by oxidative agents or the anti-fungal drug ITZ, we tested the growth of the point mutants in the presence of ITZ, H_2_O_2_ and menadione. As shown in **Figure 5B**, the MrsA^H38A^, MrsA^H96A^, and MrsA^H214A^ mutants displayed markedly decreased adaptation to the azole ITZ (0.75 μg/ml), showing a phenotype similar to that of the *mrsA* deletion mutant, with very severely disrupted colony formation. In comparison, the MrsA^G60A^ mutant showed a phenotype similar to that of the parental wild-type strain and the *mrsA*-reconstituted strain. These data suggest that the three conserved histidine residues are closely related to the azole antifungal stress response. Similar to the situation induced by ITZ, site-directed mutation of the conserved histidine (His) residues of MrsA resulted in more severe growth defects in medium supplemented with H_2_O_2_ or menadione than were observed in the parental wild-type strain or in the non-conserved site-directed control mutant MrsA^G60A^ (**Figure 5B**). Taken together, our data demonstrate that the three conserved His residues of MrsA located at positions 38 (H1), 96 (H2), and 214 (H5) that are related to iron transport in yeast are also required for resistance to plasma membrane stress induced by ITZ and for resistance to oxidative stress induced by H_2_O_2_ or menadione in *A. fumigatus*.

### MrsA Is Crucial for Virulence in a Murine Model of Invasive Aspergillosis

**FIGURE 6:**
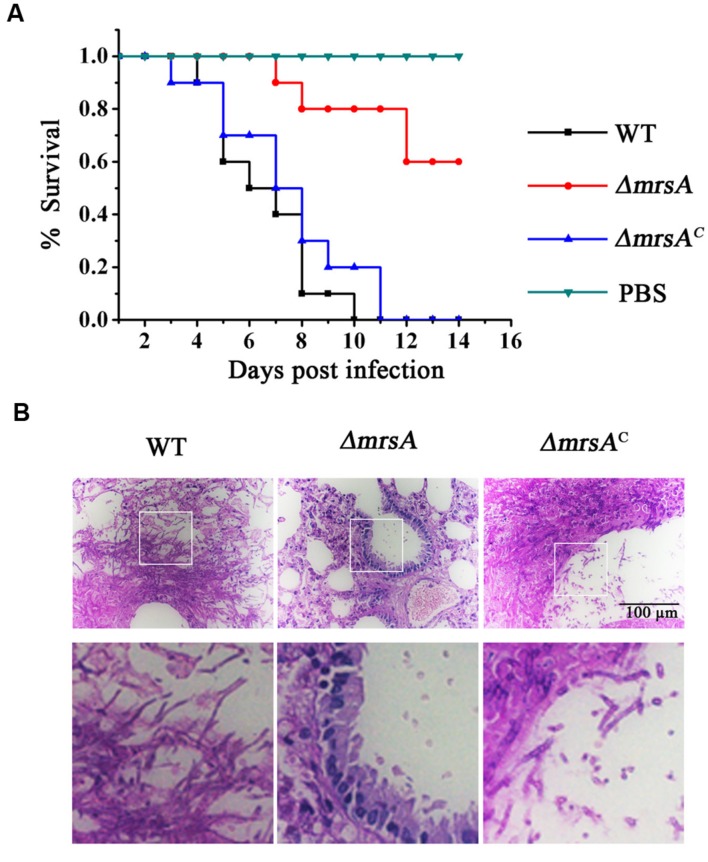
**Virulence of *ΔmrsA* in a murine model of invasive pulmonary aspergillosis. (A)** Survival curve of mice that infected with wild type, *ΔmrsA*, *ΔmrsA^C^* and PBS. **(B)** Histopathological analyses were conducted using lung tissue isolated from the mice that sacrificed at the same day post infection with each strains. Periodic Acid–Schiff (PAS) stains was utilized to visualize fungal growth.

To evaluate the virulence of the *ΔmrsA* mutant, we compared the difference of virulence in the parental wild-type, *ΔmrsA* mutant, and *ΔmrsA^C^* strains in an immunocompromised murine lung infection model. Mice infected with parental wild type or *ΔmrsA^C^* strain began to die at days 3 and 4 respectively post-inoculation while the mice infected with *ΔmrsA* strain began to die at day 7. Until day 10–11, all mice infected with parental wild type or *ΔmrsA^C^* died while the mice infected with *ΔmrsA* still displayed 60% survival until day 14. As shown in the survival curves in **Figure 6A** both parental wild type and *ΔmrsA^C^* caused high mortality with no significantly difference by log-rank analysis (*p* = 0.447). In contrast, the mortality rate of mice that infected with *ΔmrsA*, was significantly lower than that of the parental wild type (*p* < 0.0001) and *ΔmrsA^C^* strain (*p* < 0.0001). To check whether the mice that sacrificed had the infection of *A. fumigatus*, histopathological examinations of lung sections was performed. As shown in **Figure 6B**, histopathological examinations revealed that lungs from mice that inoculated with parental wild type or the reconstituted strain displayed aggressive fungal growth, which intruded into the pulmonary epithelium around lung airways. In contrast, no apparent colonies or fungal growth was detected in the lungs that infected by *mrsA* null mutant, which suggesting that the host immune system was able to eliminate the conidia of *mrsA* null mutant. Overall, histopathological analysis combined with survival curve strongly suggests that MrsA is required for *A. fumigatus* virulence.

## Discussion

For many opportunistic pathogens, the ability to obtain iron is important for growth and virulence during infection of its host (Jung et al., 2006; Haas, 2012). Mitochondria, the major consumers of cellular iron, play significant roles in the balance of cellular iron. In this study, we identified MrsA, a homolog of the yeast mitochondrial iron transporter ScMrs4p, in *A. fumigatus* and showed that it functions in regulating cellular iron homeostasis. The evidence to support MrsA as a putative mitochondrial iron transporter is as follows: first, GFP-tagging experiments showed that MrsA is localized in mitochondria (**Figure 1A**). Second, BLASTP analysis revealed that MrsA possesses a conserved MCF domain with great similarity to the MCF domain of its putative homologs. MrsA contains six conserved transmembrane helices (H1–H6) and exhibits a tripartite structure related to mitochondrial iron transport (Supplementary Figure S1). Third, deletion of *mrsA* severely affected colony growth both under conditions of iron depletion and under conditions of iron excess (**Figure 1C**). Finally, when either the *mrsA* gene or the previously identified yeast mitochondrial iron transporter gene *Scmrs4* was used to complement the *ΔmrsA* strain, the *ΔmrsA* defect observed under iron depletion and iron excess conditions was fully rescued, indicating that the defect is specifically due to loss of *mrsA* and that *mrsA* is a functional homolog of *Scmrs4* in *A. fumigatus* (**Figure 1C**). Taken together, these findings suggest that the putative mitochondrial iron transporter MrsA plays an important role in colony growth by regulating the balance of cellular iron in *A. fumigatus*.

Mitochondrial iron is of importance for many cellular processes. Our data indicated that deletion of *mrsA* in *A. fumigatus* is not lethal either during normoxic or hypoxic conditions (1% O_2_), suggesting that possibly there are additional mitochondrial iron transporters. In *S. cerevisiae*, the mitochondrial transporter Rim2 (Mrs12) has been shown to co-import pyrimidine nucleotides and iron (Yoon et al., 2011). Overexpression of *rim2* was able to rescue the iron-related defect phenotype of *Δmrs3Δmrs4*, while deletion of *rim2* impaired Fe–S protein maturation, demonstrating Mrs3/4p independent mitochondrial iron import (Yoon et al., 2011; Froschauer et al., 2013). Therefore, it is likely that *A. fumigatus* also possesses MrsA independent mitochondrial iron import.

### Possible Mechanism of the Disrupted Cellular Iron Balance in *ΔmrsA*

In *A. fumigatus*, it has been shown that cellular iron homeostasis is controlled by two central transcription factors, SreA and HapX (Schrettl and Haas, 2011). Under conditions of iron sufficiency, SreA is activated and represses the expression of genes related to iron acquisition, including the genes associated with the two high-affinity iron uptake systems RIA and SIA. In contrast, during iron starvation, enhanced expression of HapX activates the SIA iron uptake system and inhibits the activity of the iron-consuming pathway (Schrettl and Haas, 2011).

Here, we showed that deletion of *mrsA* significantly decreased the ability of *A. fumigatus* to adapt to either iron-limiting or iron excess conditions, suggesting that MrsA affected colony growth by regulating cellular iron homeostasis (**Figure 1C**). To confirm this hypothesis, we first tested the mRNA levels of the two central transcription factors, *sreA* and *hapX*. Interestingly, the mRNA abundance of *sreA* was significantly decreased in *ΔmrsA* compared to the parental wild type, whereas *hapX* mRNA levels showed no detectable change (**Figure 2**). This phenomenon is consistent with previous reports that deletion of *sreA* de-represses (activates) both RIA and SIA (Oberegger et al., 2001; Schrettl et al., 2008). Therefore, it is possible that the primary reason for the disrupted cellular iron balance in *ΔmrsA* was a defect in SreA expression induced by deletion of *mrsA*.

### Deletion of *mrsA* Induces Abnormal Cellular ROS Accumulation and Hypersusceptibility to Antifungals

Currently, many lines of evidence from the study of fungal pathogens have revealed that mitochondrial dysfunction usually leads to changes in susceptibility to antifungal drugs (Shingu-Vazquez and Traven, 2011). The data presented in this work demonstrate that the putative mitochondrial iron transporter MrsA of *A. fumigatus* is required for fungal cell responses to treatment with the azole antifungal ITZ and that *ΔmrsA* cells show a hypersusceptibility phenotype.

To identify the reason for the increased sensitivity of *mrsA* mutants to the antifungal ITZ, we examined the intracellular accumulation of antifungal drugs and the cellular content of the drug-targeted substrate ergosterol. These two factors have been identified as two main mechanisms of drug susceptibility in a wide variety of fungal pathogens (Wei et al., 2015). Unexpectedly, neither cellular ergosterol content nor the intracellular accumulation of antifungal drugs differed significantly in the *ΔmrsA* and the parental wild-type strains (**Figures 4C,D**). Interestingly, when treated with ITZ in combination with the antioxidant L-ascorbic acid sodium (Vc) or iron, the defective colony phenotype of *ΔmrsA* was almost completely restored to the normal colony phenotype of the parental wild type strain (**Figure 4E**). On one side, L-ascorbic acid sodium is a commonly used antioxidant that can interact with ROS to alleviate oxidant damage to the organism; on the other side it improves iron uptake by reducing iron and fueling low-affinity iron uptake (Eisendle et al., 2003). It is possible that improved iron uptake induced by L-ascorbic acid sodium could reduce the ROS production through activating activities of superoxide dismutase (SOD) and catalases. Our data clearly suggest that the increased sensitivity of *ΔmrsA* to ITZ may be mainly due to the increased ROS levels present in cells. Indeed, as shown in **Figure 3C**, we detected increased production of ROS in *ΔmrsA* compared to the parental wild type. In *C. albicans* and *S. cerevisiae*, it also has been demonstrated that increased ROS production triggered by the fungicide miconazole contributes to its antifungal activity (Kobayashi et al., 2002; Belenky et al., 2013). Moreover, previous studies suggest that increased ROS production can damage DNA or block DNA-repair pathways, resulting in cell death (Belenky et al., 2013). Collectively, our data, together with results from previous studies in yeasts, indicate that ITZ susceptibility is closely related to ROS production.

### Three Conserved Histidine Residues Related to Iron Transport in MrsA Are Required for Response to Oxidative and Azole Stresses

Given the crucial role of MrsA in cellular iron homeostasis and the stress response, we dissected the molecular characteristics of this role. On the basis of information from *S. cerevisiae* homologs Mrs3p and Mrs4p, three important histidine residues of MrsA were identified (**Figure 5A**). The results showed that all three identified histidine residues are essential in both iron acquisition and in the stress responses induced by oxidant and ITZ (**Figure 5B**). In yeasts, putative structures of ScMrs3p and ScMrs4p were proposed based on the information from the bovine nucleotide carrier of the MCF family (Brazzolotto et al., 2014). In this proposed structure, the transport of iron into mitochondria is mediated by a switch between two different conformations. The structural information suggests that the three conserved histidine residues of Mrs3p located at positions 48, 105, and 222 faced to the internal cavity of the transporter, which is accessible from the intermembrane space. The spatial arrangement of histidines, especially His48 and His105, which form a structure resembling a spiral staircase, could allow iron ions to move to the bottom of the cavity when the carrier changes conformation (Brazzolotto et al., 2014). Based on the structural features of ScMrs3p and ScMrs4p and the phenotypes of the histidine mutants of MrsA, we infer that the histidine residues located at positions 38, 96, and 214 are crucial for MrsA-mediated mitochondrial iron transport.

Because mitochondria represent the major site at which ROS are generated, the dynamic equilibrium of ROS generation, especially in the detoxification of ROS, is very important in avoiding ROS-related cell damage (Kandola et al., 2015). Notably, detoxification of ROS is mainly catalyzed by SOD and catalases, enzymes that contain iron or iron-cluster-dependent components (Mittra et al., 2016). Moreover, in *S. cerevisiae*, Mrs3p and Mrs4p also are capable of transporting copper into mitochondrial (Vest et al., 2016). Copper, however, plays crucial roles in regulating the activity of SOD. Therefore, it is also possible the defective putative mitochondrial iron transporter MrsA most likely produces a reduction in mitochondrial iron and copper or iron and copper cluster content, thus impairing the activity of SOD or catalases and increasing ROS levels. In contrast, the antioxidant L-ascorbic acid sodium rescues the colony defects of *ΔmrsA* induced by ITZ, H_2_O_2_ or menadione (**Figures 3D** and **4E**). Taken together, our data indicate that three conserved histidine residues in MrsA that are related to iron transport are also required for cellular responses to oxidative and azole stressors that may mediate the production of endogenous ROS.

### MrsA Is Required for the Virulence

Our data (**Figure 6**) demonstrate that deletion of the mitochondrial iron transport MrsA results in significant attenuation of virulence of *A. fumigatus* as previously shown for the homologs of *C. albicans* and *L. amazonensis* (Xu et al., 2014; Mittra et al., 2016). The *in vitro* histopathological analysis suggests that the attenuated virulence of *mrsA* null mutant might result from its hypersensitive to oxidative stresses since the survival and the escape of pathogen conidia from macrophage killing of the host partly depend on the release amount of host ROS. In addition, the decreased competition ability for other nutrition components from the host might be another reason since we found that *mrsA* deletion caused a very sick colony phenotype on minimal media and particularly during iron starvation (**Figure 1C**), which is in agreement with the upregulation of MrsA at the protein level during this condition (**Figure 1B**). Notably, iron starvation has been shown to be crucial for virulence of *A. fumigatus* (Haas, 2012).

.

## Conclusion

Our study strongly suggests that MrsA plays important roles in response to oxidative and azole stresses by affecting the ROS level in *A. fumigatus* through the regulation of cellular iron homeostasis. MrsA is required for full virulence and azole resistance of *A. fumigatus* and the conserved His residues of MrsA play important roles in the function of MrsA homologs. However, MrsA is not a good antifungal drug target candidate since this protein is relative conserved in eukaryotic system.

## Author Contributions

Conception and design of the investigation and work: NL, SZ, and LL. Completion of the experiments: NL, XX, and HQ. Evaluation and analysis of the results: NL and XX. Manuscript writing: NL and LL. Final approval of manuscript: NL, XX, HQ, SZ, and LL.

## Conflict of Interest Statement

The authors declare that the research was conducted in the absence of any commercial or financial relationships that could be construed as a potential conflict of interest.
