# Supplementary material for: A Putative Mitochondrial Iron Transporter MrsA in Aspergillus fumigatus Plays Important Roles in Azole-, Oxidative Stress Responses and Virulence
Source: Front Microbiol. 2016 May 12;7:716. doi: 10.3389/fmicb.2016.00716 (PMC4922219; doi:10.3389/fmicb.2016.00716)
Supplement: Supplementary file 1 [file Data_Sheet_1.DOCX]

Supplementary Material

**A putative mitochondrial iron transporter MrsA in *Aspergillus fumigatus* plays important roles in azole-, oxidative stress responses and virulence**

Nanbiao Long, Xiaoling Xu, Hui Qian, Shizhu Zhang, Ling Lu*

*Corresponding author: Ling Lu: linglu@njnu.edu.cn


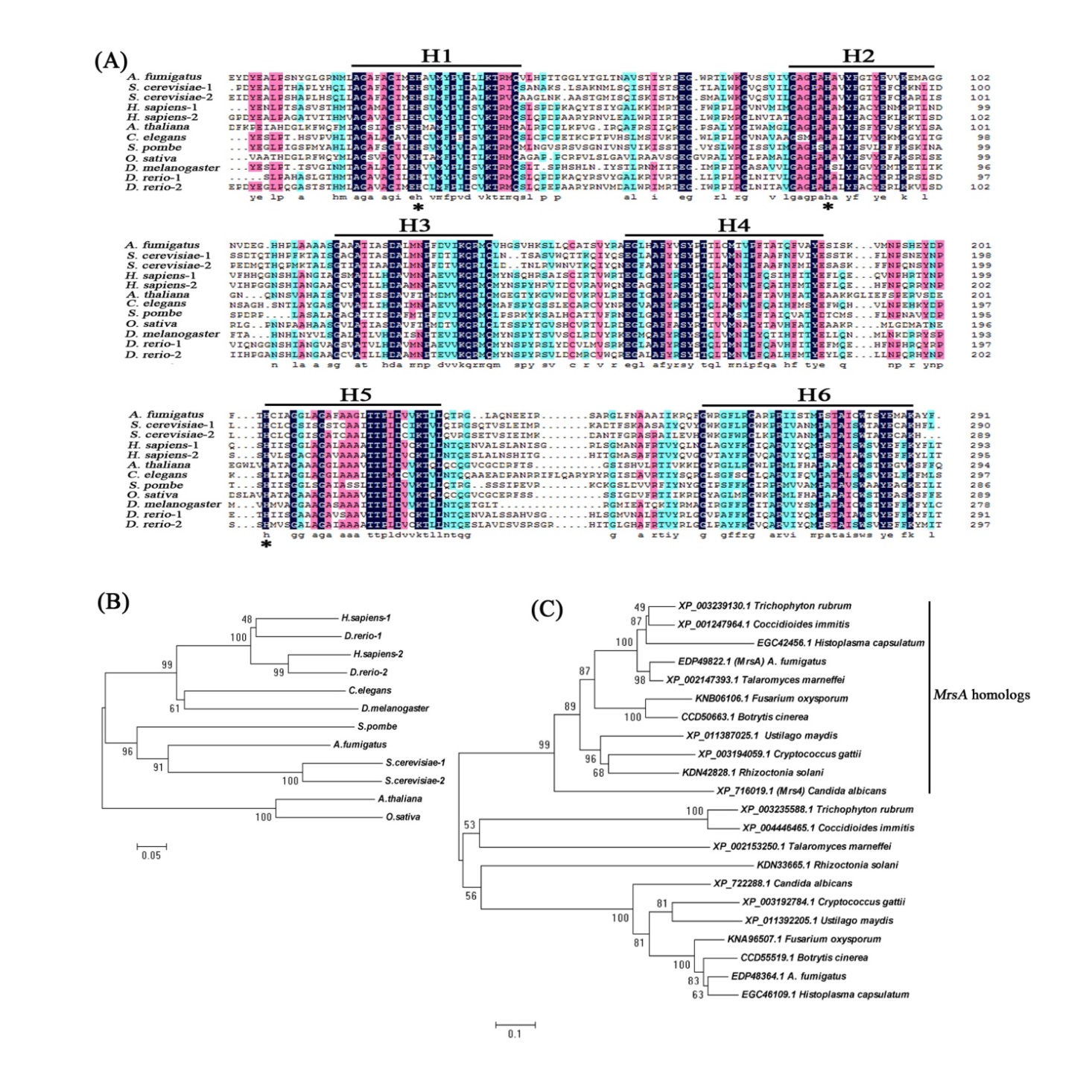


**Supplementary Figure 1.** Bioinformatics analysis of MrsA. (A) Sequence analysis of MrsA homologs of selected organisms. The blue areas represent identical conserved amino acid residues; H1-H6 represent the six transmembrane helices. In H1, H2 and H5, the highly conserved histidine residues in the mitochondrial iron transporter are marked by asterisks (*). (B) Phylogenetic analysis of MrsA homologs from selected fungi, animals and plants. (C) Phylogenetic analysis of MrsA homologs from selected pathogenic fungi.


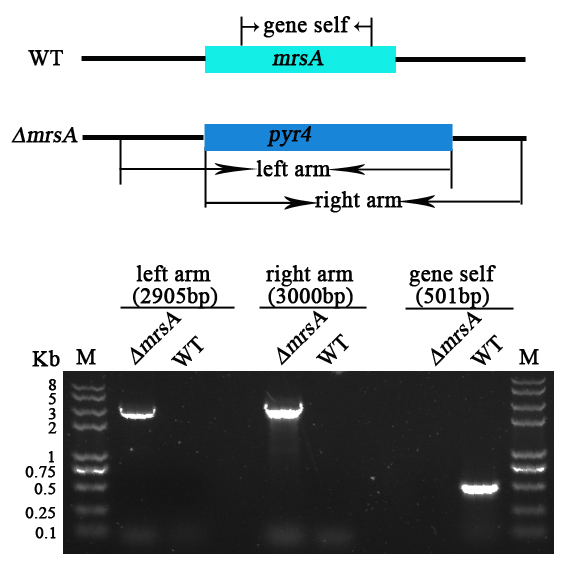


**Supplementary Figure 2.** Deletion of *mrsA* was confirmed by diagnostic PCR. The left arm (2905 bp) and right arm (3000 bp) were used to verify homologous replacement of *mrsA* by the *pyr4* marker. Gene self (501 bp) was used to confirm that the ORF of *mrsA* was completely knocked out.

**
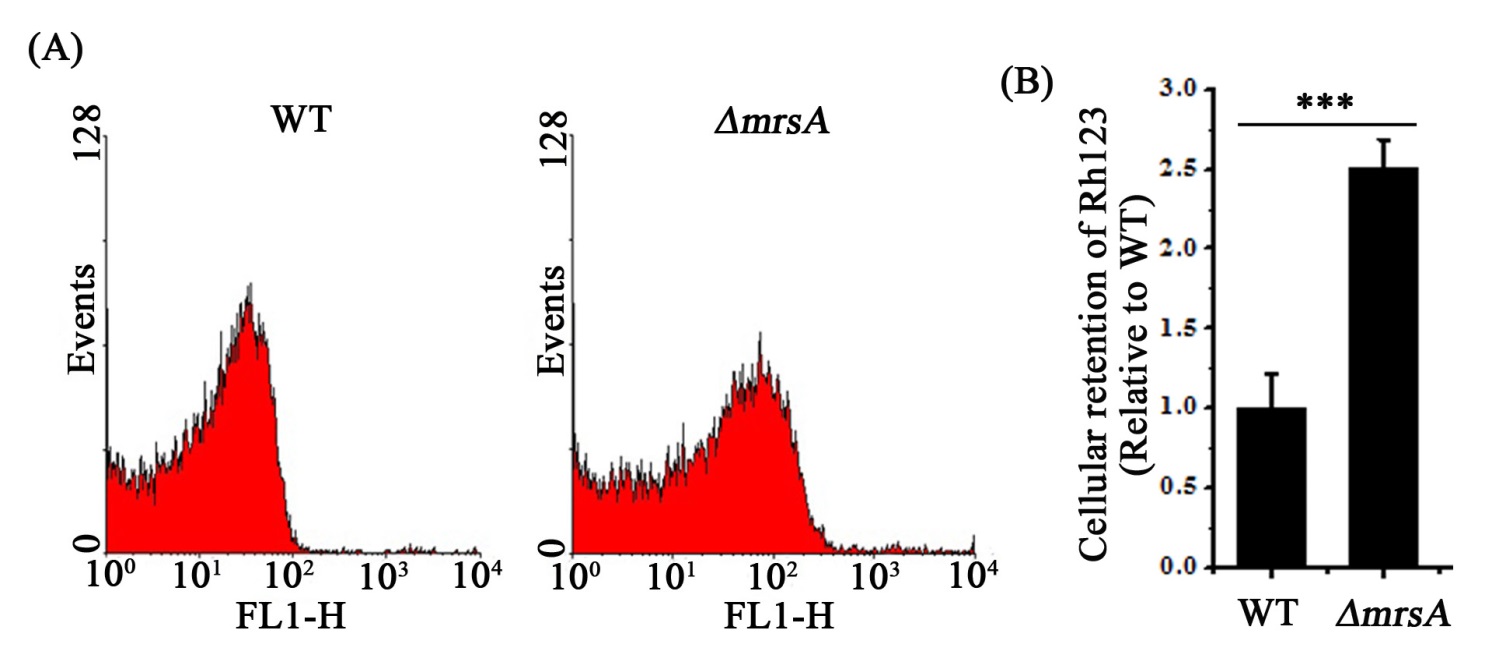
**

**Supplementary Figure 3.** A. Rh123 accumulation is increased in *ΔmrsA*. In this experiment, 10^7^ conidia per ml of the parental wild-type and *ΔmrsA* strains were cultured at 37°C for 4 h in YAG medium; the medium was then supplemented with Rh123 (10 μmol) at 37°C for 1 h. Rh123 accumulation was measured by flow cytometry. B. Retention of Rh123. ***P< 0.001 compared with the parental wild type.

**Supplementary Table 1.** Primers used in this study.

| Primer name | Primer sequence 5' - 3' |
| --- | --- |
| mrsA P1 | TACATTCCGTCTCACCGTATTCA |
| mrsA P2 | TCAATCCATCGGCAAAAGTAG |
| mrsA P3 | CGATTAAGTTGGGTAACGCCATGTTCCGGCCAAGGCCATAGTTTGA |
| mrsA P4 | ATAAGTAGCCAGTTCCCGAAAGCGCCAGGAGTGAGACTTTCGAG |
| mrsA P5 | ATTAATAACCGCAGGGAAAGC |
| mrsA P6 | CTACCCCACGGTGATACTACGA |
| mrsA S1 | CCTGGAAATAGTCATCAAGCAAC |
| mrsA S2 | CAATGTCAACTCTGAAACCCTGT |
| pyr4 F | TGGCGTTACCCAACTTAATCG |
| pyr4 R | GCTTTCGGGAACTGGCTACTTAT |
| mrsA F | GCATTATCTCCCTCTCTGTTC |
| mrsA R | TGCCGCAAGTATGTCTCCAA |
| mrsA-gfp P1 | GGGATAATGGTATGTCGGCGT |
| mrsA-gfp P2 | TTTCCGTGGCGATACTGACTG |
| mrsA-gfp P3 | CCAGCGCCTGCACCAGCTCCCTCCTGGCGTTTGAAGTAGGC |
| mrsA-gfp P4 | CATCAGTGCCTCCTCTCAGACAGTGAGACTTTCGAGCTCCTACCCG |
| mrsA-gfp P5 | TCGATGGGATTGCTGATTTGA |
| mrsA-gfp P6 | GGAAGATAGAATCGATGGGCA |
| gfp-pyrG F | GGAGCTGGTGCAGGCGCTGG |
| gfp-pyrG R | CTGTCTGAGAGGAGGCACTGATG |
| hph-*Spe*I F | CGGACTAGTGAATTCCCTTGTATCTCTAC |
| hph-*Spe*I R | CGGACTAGTTCGAGTGGAGATGTGGA |
| hph F | GAATTCCCTTGTATCTCTACACACAGGC |
| hph R | TCGAGTGGAGATGTGGAGTGGGCGCTTA |
| hph-*Not*I F | ATAAGAATGCGGCCGCGAATTCCCTTGTATCTCTAC |
| hph-*Spe*I R | GGACTAGTTCGAGTGGAGATGTGGAGTGGG |
| Scmrs3 *Cla*I F | CCTTTAATCAAGCTTATCGATATGGTAGAAAACTCGTCGAG |
| Scmrs3 *Cla*I R | CTCGAGGTCGACGGTATCGATCTAATACGTCATTAGGAAATG |
| Scmrs4 *Cla*I F | CCTTTAATCAAGCTTATCGATATGAATACTTCAGAACTGTCAATAGC |
| Scmrs4 *Cla*I R | CTCGAGGTCGACGGTATCGATTCAATTTTTCATTAAAAAATGCTTAGCA |
| mrsA^H38A^ F | GGATGGGAAGGAAGCCGCTGTCATGTACCC |
| mrsA^H38A^ R | GGGTACATGACAGCGGCTTCCTTCCCATCC |
| mrsA^G60A^ F | CCTACAACAGGAGCTCTCTACACAGGCTTG |
| mrsA^G60A^ R | CAAGCCTGTGTAGAGAGCTCCTGTTGTAGG |
| mrsA^H96A^ F | CAGGTCCGGCGGCTGCAGTGTACTTCGG |
| mrsA^H96A^ R | CCGAAGTACACTGCAGCCGCCGGACCTG |
| mrsA^H214A^ F | GACCCGTTCACCGCCTGCATCGCTGGTGGCC |
| mrsA^H214A^ R | GGCCACCAGCGATGCAGGCGGTGAACGGGTC |
| RT-sreA F | CGATTTCCACTCTTCTAAACCACT |
| RT-sreA R | GAGGCTTTCAAGTTCAGACACG |
| RT-hapX F | CCGCACCATCCTTGACTTTAT |
| RT-hapX R | CGAGGTTTAGGCAAGGTATGAA |
| RT-sidA F | AACACTTGAGACCTACGGGACA |
| RT-sidA R | TTACAACCTTGAAGCCAGATGC |
| RT-mirB F | AGCTTGATCTATTCGTCCCTCC |
| RT-mirB R | CCCTTGGTCTGAGTCATGTTTT |
| RT-ftrA F | GACAAACTCGGCAACCTAT |
| RT-ftrA R | CGTGCTGACCTCGACATCT |
| RT-fetC F | ACAACGACGATGCTGGAAA |
| RT-fetC R | CCTGGAAACGGAGGACAAA |
| RT-cycA F | GCCTACACCGACGCCAACA |
| RT-cycA R | CGCCTGCAATGCCAACCAT |
| RT-tubA F | ACGTTACCTCACCTGCTCTGC |
| RT-tubA R | GATGTTGTTGGGAATCCACTCA |
